# Attributes of primary health care in Mato Grosso do Sul state: PCAT-Brazil paired for users and health professionals, 2018

**DOI:** 10.1186/s12913-022-08363-x

**Published:** 2022-07-29

**Authors:** Rafael Aiello Bomfim, Hazelelponi Querã Naumann Cerqueira Leite, Edilson José Zafalon, Alessandro Diogo De-Carli, Mara Lisiane de Moraes dos Santos

**Affiliations:** 1 School of Dentistry, Federal University of Mato Grosso do Sul, Campo Grande, Brazil; 2 Integrated Institute of Health, Federal University of Mato Grosso do Sul, Campo Grande, Brazil

**Keywords:** Primary health care, Public health, PCAT-Brazil

## Abstract

**Objective:**

The objective of the present study was to analyse the quality of adults and older adults health care in Primary Health Care (PHC) services in the State of Mato Grosso do Sul, 2018.

**Methods:**

A quantitative survey was carried out in which the municipalities participating in the study included the four macro-regions following the Director Regional Plan (DRP). In this study, the quality of care was verified using the validated version of the PCAT-Br for adult and older adults users over 18 years of age and professionals. The professional’s and users’ views were compared between PHC attributes in the State of Mato Grosso do Sul. We performed the paired student t-test. STATA v.14.2 software (College Station, TX, USA) was used for the analyses. Sensitivity analysis was done to compare socio-demographic characteristics.

**Results:**

Eight hundred twenty-five users and 424 professionals participated in the study. According to users, the Accessibility attribute had the worst performance in all macro-regions (mean score PCAT = 3.58). There were significant differences between the perception of users and professionals (PCAT = 5.32 for users and PCAT = 7.11 for professionals) in all attributes evaluated.

**Conclusions:**

There was a difference in users’ and professionals’ perceptions between PHC attributes. Therefore, it is necessary to strengthen PHC care networks in the State, mainly considering the users’ perspectives.

## Introduction

In Brazil, Primary Health Care (PHC), through the work of Family Health teams (eSF), corresponds to the structuring and coordinating axis of the Unified Health System (SUS) [1], being the main access route for users to health services. SUS services, whose actions must be centred on the individual, meeting their health needs [2]. Shreds of evidence [3–6] indicate the positive impact of PHC on the primary health indicators in the country after implementing the Family Health Strategy (ESF), mainly due to the significant increase in the number of eSF, implying greater coverage of this service in Brazilian territory. From this perspective, the power of PHC is demonstrated as a policy that supports access to health care for a significant portion of the Brazilian population, an issue that becomes critical when considering the continental dimensions and the pronounced Brazilian socioeconomic inequality.

However, the country still faces difficulties implementing PHC and the quality and effectiveness of the services offered [7–11]. The organisation and orientation of services by PHC attributes promote better indicators, greater user satisfaction, equity, lower costs, and, consequently, positively impact the health of the population [1, 12]. The evaluation of operational quality in PHC is linked to the presence and extent of four essential attributes and three derived ones [12]. The essential ones correspond to attention to the first contact (accessibility), longitudinally, comprehensiveness and care coordination, and the derived attributes consist of people- and family-centred (family orientation) health care, community orientation and cultural competence [12].

In this sense, the orientation of the work performed by professionals to fulfil PHC attributes has been widely performed in the world [13–23] and Brazil [24–27] through the use of the Primary Care Assessment Tool (PCA-T) [12]. Therefore, to the extent that PHC services are strongly oriented towards achieving the presence of most of these attributes, their ability to provide comprehensive care is considered optimized [27].

Thus, it is important to use strategies to consolidate universal and equitable access within the scope of the SUS through tools that promote evaluation and monitoring of the performance of PHC teams [12]. The evaluation process also makes it possible to unveil the experiences lived by both users and professionals that can reflect on other levels of care services in the health care network and other experiences articulated with the rest of the system [12]. Given the different organisational arrangements and specific aspects of care offerings in health services existing in Brazil, the search for performance evaluation, knowledge of the opinions of professionals who work on it, as well as information on users’ experiences, can evidence the actual effectiveness of PHC and assist in the definition of public policies [12, 28].

Although the evaluation of PHC through the PCATool is a topic widely explored by researchers around the world [20–23], there are limited publications that analyse and problematise, at the same time, the set of essential and derived attributes of PHC services from the perspective of users and professionals, comparing them. Such approaches are essential, considering that one study [29] has shown discrepancies between the views of users and professionals about the health services offered, with professionals tending to evaluate services more positively concerning users. Additionally, the PCATool proposal is to identify whether PHC services are guided by the set of their attributes, whose presence and extent reflect in health indicators, user satisfaction, costs and equity [30] and, through good results, promote comprehensive care [14] with impacts and positive effects on the health of people and populations.

The objective of this study was to analyse the quality of adult health care in PHC services from the perspective of users and professionals in the four health macro-regions of the State of Mato Grosso do Sul, using the PCAT-Brazil instrument.

## Methods

**Fig. 1:**
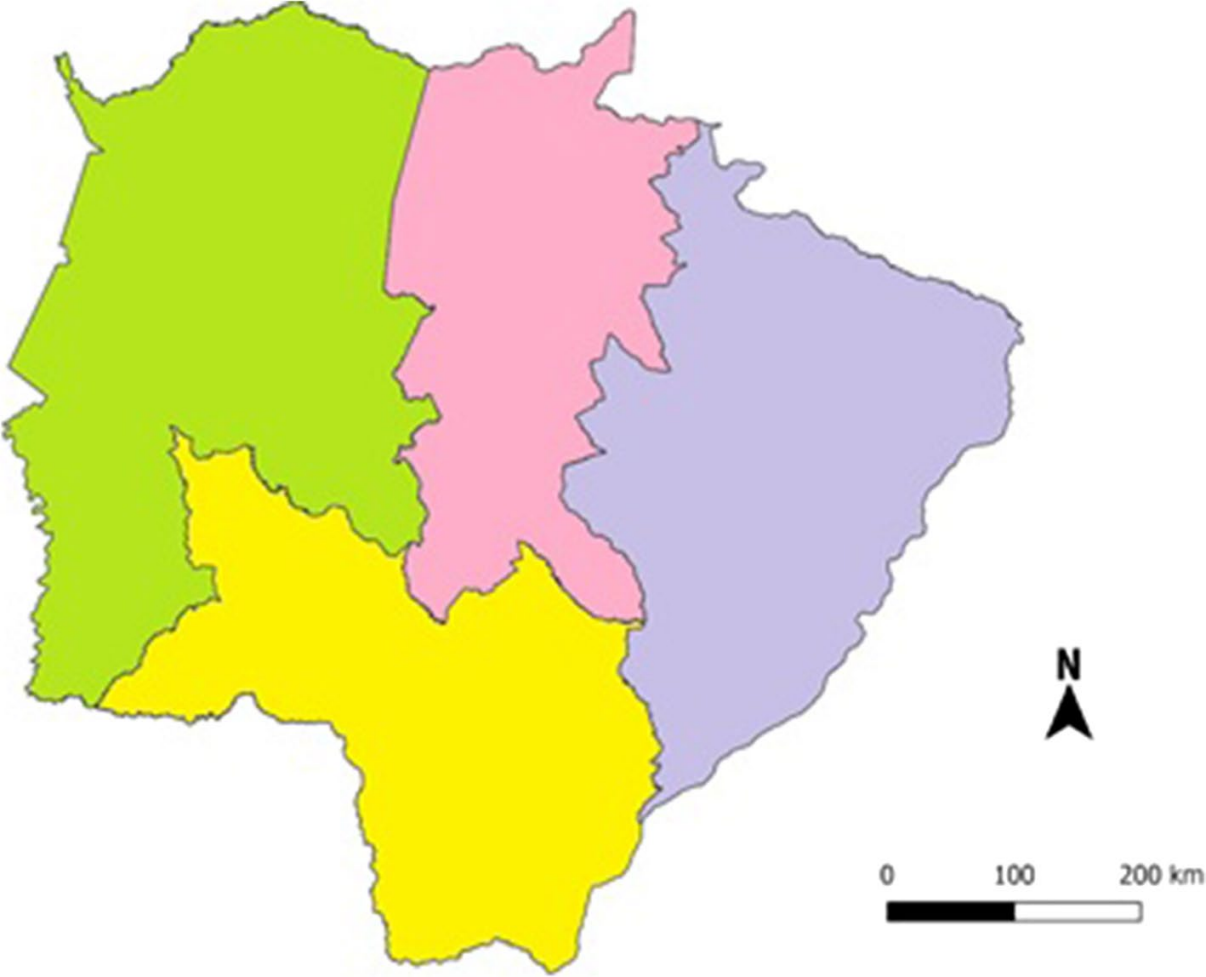
Distribution map of the macro-regions of the State of Mato Grosso do Sul

This study was a cross-sectional research carried out from August to December 2018 in Mato Grosso do Sul State. Mato Grosso do Sul is an important Brazilian state in the Midwest region and is responsible for a strong agribusiness that supplies the other geographical regions in the country. In Brazil, agribusiness accounts for approximately 25% of the GDP (Gross Domestic Product) [31]. The State of Mato Grosso do Sul is one of the 27 federative units in Brazil, located in the south of the Central-West Region, divided into 79 municipalities and occupies an area comparable in size to Germany, with a population of 2,839,188 inhabitants with an average of HDI 0.729 [31]. As in all of Brazil, health promotion and prevention for the population of the State of Mato Grosso do Sul is offered to every Brazilian citizen through the Unified Health System (SUS) with full, universal and free access to health services [31]. The municipalities participating in the study included the 11 health microregions of Mato Grosso do Sul following the Regional Master Plan (RMP), which divided the State into four macro-regions: 1) Campo Grande, Dourados, Três Lagoas and Corumbá (Fig. 1).

### Sample size calculation

The sample size calculation was performed on the representativeness of the macro-regions using the STATA software (College Station, TX, USA). The total number of health professionals who worked in the Family Health Teams (as professionals who passed public examinations and are not on a temporary contract) was considered, representing all macro-regions. The parameter used to perceive differences in the mean scores of 0.25 between users and professionals, considering all attributes in the PCAT-Br instrument with an alpha of 5%, power of 80%, and allocation of 2 users for one professional. Our sample size estimated 800 users and 400 PHC professionals. Assuming 10% of losses, our final sample size was estimated at 880 users and 440 professionals. Thus, the municipalities were randomly selected, and their distribution was proportional to the number of municipalities per macro-region. The participating municipalities were defined by random selection, including all the microregions. Therefore, we respect at least four health units in each municipality and four users per health unit selected to obtain an overview of the perception of users and professionals (ratio 2:1) of the SUS in the State of Mato Grosso do Sul.

### Calibration of examiners

Data collection was performed by interviewers who received theoretical and practical qualifications (24 hours) and underwent inter-and intra-examiner calibration, resulting in a Kappa index equal to or greater than 0.87. Then, the items and questions of the Primary Care Assessment Tool (PCAT-Brazil), validated in Brazil [25, 26], were studied and discussed to fluency the execution. The possible doubts that arose were resolved in time to ensure the reliability of the data. We chose this instrument because it has already been used in several countries, which gives it the characteristic of international comparability [13–23].

### Data collection

In this study, the quality of care was verified using the PCAT-Brazil instrument for adult users over 18 years of age and professionals [25]. This instrument measures the presence and extent of essential and derived attributes of PHC and thus infers strong or weak orientation for PHC of the evaluated service [32]. In addition, the municipal health departments indicated the Primary Health Units (PHU) and those that accepted and agreed to participate in the research were included. However, the health units indicated by the secretariats were restricted because they were made up of Family Health teams, which helped in the logistics of data collection for the research.

The sample of health professionals consisted of those in the eSF in the selected units. In addition, professionals should assist adult users, fulfil a workload of at least 20 hours per week and work at the health unit for at least one year. Exclusion criteria were limited to professionals who cared for children, worked at the health unit for less than a year and were temporary professionals. The sample of professionals was determined by random selection, as those who worked in the health units indicated by the municipal secretariats were interviewed. After including professionals, we included only users attended by the included professional. The interviewers collected data from the users at the participants’ homes. These were randomly selected from a list also provided by the municipal health departments, containing the families assigned to each area of activity of the ESF participating in the study. Users should have resided in the territory for at least one year for the inclusion criteria.

### PCAT-Brazil score

The scores for each attribute or component were calculated by the simple arithmetic mean of the response values of the items that make up each attribute or component. Then, the scores were calculated and transformed into a scale from 0 to 10, according to the PHC Assessment Instrument Manual (PCAT-Brazil) [32].

In the adult version, answered by users, the essential score is measured by the sum of the average score of the components that belong to the essential attributes (plus Affiliation Degree) divided by the number of components. The overall score is measured by the average score of components belonging to essential attributes plus components belonging to derived attributes (plus Affiliation Degree), divided by the total number of components. More information on calculation scores could be seen elsewhere [32].

### Outcome variable

The score for each essential and derived PHC attribute was the dependent variable. The methodology of the PCAT instrument considers a score ≥ 6.6 as a minimum quality value to assess primary care services from the adult user’s perspective, and in both surveys, this general quality value [27].

### Main exposures variables

The main exposure variable was the professional/user data.

### Descriptive variables

Descriptive data from users like gender, income, education, self-reported race, according to the Brazilian IBGE, time living in the region, sex and age group were reported. Furthermore, descriptive data from professionals like the category of the health professional, gender, and if they lived and worked in the same city and time since graduation was described.

### Statistical analysis

To compare the averages between professionals and users, paired Student’s t-test was used (the professional was paired with the users). A significance level of 5% was considered, and Data Analysis and Statistical Software (STATA) v.14 (College Station, TX, USA) was used. In addition, chi-squared tests between each socio-demographic characteristic were done as a sensitivity analysis to show that it did not influence variations in the Overall Score.

The Research Ethics Committee of the Federal University of Mato Grosso do Sul approved this study (CAAE 58735316.4.0000.0021).

## Results

A total of 825 PHC users from 29 municipalities in the State of Mato Grosso do Sul participated in the study. Therefore, we had 6,25% participant losses as the estimated sample was the same as the included sample. Regarding the socio-demographic profile of the participants, female users were predominant, in the age groups of 18 to 34 years, self-declared whites and Browns, with an elementary school and above ten years resided in the area assigned to the health units, as shown in Table 1.

**Table 1:** Descriptive characteristics of all users, Mato Grosso do Sul State, Brazil (*n* = 825)

Socio-demographic variables	n	%
**Gender**		
Female	686	83.2
Male	139	16.8
**Self-Declared Race**		
Whites	299	36.2
Browns	335	40.6
Blacks	45	5.5
Indigenous	7	0.8
Yellow	3	0.4
Missing	136	16.5
**Income**		
up to 1 MW	246	29.8
between 1 and 2 MW	172	20.8
between 2 and 3 MW	53	6.4
between 3 and 5 MW	17	2.1
between 5 and 8 MW	1	0.1
up to 8 MW	1	0.1
do not know	194	23.5
Missing*	141	17.1
**Schooling**		
illiterate	46	5.6
1–4 years	325	39.4
4–8 years	249	30.2
> 8 years	63	7.6
Post-graduation	7	0.8
Missing	135	16.4
**Time of residence in the city**		
Up to 10 years	245	29.8
above ten years	433	52.6
Missing	147	17.9
**Age**		
18 a 34	244	29.6
35 a 44	142	17.2
45 a 59	194	23.5
Over 60	110	13.3
Missing	135	16.3

*MW* Minimum wage

* Some participants do not agree on report socio-demographic data. This is why we had missing information on demographic data

Concerning the profile of the 424 professionals linked to PHC in the 29 municipalities of the State of Mato Grosso do Sul, the majority did not reside in the region attached to the health unit they operate. We had 3,63% on professionals losses. Furthermore, they presented a predominant age between 22 and 44 years, trained for more than ten years and predominantly nurses, as shown in Table 2.

**Table 2:** Descriptive characteristics of health professionals in Mato Grosso do Sul State, Brazil (*n* = 424)

Variable	n	%
**Gender**		
Female	283	66.7
Male	77	18.2
Missing	64	15.1
**Health Professional**		
Nurses	176	41.5
Dentists	128	30.2
GP (General Practitioners)	56	13.3
Other	64	15.0
**Live in the same city that works.**		
No	19	83.7
Yes	355	4.5
Missing	50	11.8
**Age**		
22–34	163	38.4
35–44	112	26.4
45–59	67	15.8
> 60	20	4.7
Missing	62	14.6
**Time since graduation**		
up to 10 years	170	40.6
Above ten years	190	44.8
Missing	62	14.6
**Macroregion**		
Campo Grande	215	50.7
Dourados	144	34
Três Lagoas	52	12.3
Corumbá	13	3

The results referring to the attributes of people- and family-centred (family orientation) health care differed between the participants. The scores were higher for professionals in all macro-regions than users (Table 3). The average score for family orientation ranged from 4.72 in the macro-region of Campo Grande to 6.58 in the region of Corumbá. The highest average score related to people orientation was equivalent to the Três Lagoas macro-region with 5.34. There were significant differences in all analysed attributes between professional and user views, being the views about users under the minimum quality value of 6.6.

**Table 3:** Distribution of scores under Professionals and users in Mato Grosso do Sul (*n* = 1249).

Atributo	Category	Mean	SD	CI(95%)	p
Accessibility	Professionals	3.94	1.31	3.81–4.06	< 0.001
	Users	3.58	1.66	3.46–3.69	
Longitudinally	Professionals	7.7	1.42	7.56–7.83	< 0.001
	Users	6.39	2.2	6.24–6.54	
Coordination - integration of care	Professionals	7.49	1.78	7.32–7.66	< 0.001
	Users	2.82	3.81	6.24–6.54	
Coordination - health systems	Professionals	8.46	1.71	8.30–8.63	< 0.001
	Users	6.89	2.3	6.73–7.05	
Integrality - Disposable services	Professionals	7.1	1.49	6.96–7.24	< 0.001
	Users	5.69	1.52	5.59–5.79	
Integrality - Presting services	Profissionais	6.63	2.26	6.41–6.84	< 0.001
	Users	4.1	2.53	3.92–4.27	
Family Orientation	Professionals	8.57	1.96	8.38–8.75	< 0.001
	Users	5.33	3.24	5.11–5.56	
Community Orientation	Profissionais	7.03	2.09	6.83–7.23	< 0.001
	Users	4.48	2.51	4.31–4.66	
General Score	Professionals	7.11	1.05	7.01–7.21	< 0.001
	Users	5.32	1.46	5.22–5.42	

*SD* Standard Deviation, *CI* Confidence Interval

Sensitivity analysis showed no differences between socio-demographic data and the four macro-regional in Mato Grosso do Sul State (*p* > 0.05).

## Discussion

This study highlighted one critical finding. It was the difference in perception between users and professionals in all analysed attributes, with worse evaluations for all attributes among users, not reaching the minimum of 6.6. 

The PHC acts as a care coordinator for the population to have population benefits [1]. The present research results showed significant differences in the evaluations of PHC attributes between users and professionals. We observed a weak health services orientation evaluated from the users’ experience and perspective and a strong orientation from the professionals’ point of view. These data confirm the results of previous studies that used the PCAT-Brazil [33–37]. The overestimated perception from professionals regarding the evaluation of the service is favourable.

Nevertheless, the confrontation of ideas between professionals and users shows important differences. The highest scores best evaluated by both group participants converge to the attribute care coordination - Information System, corroborating the results of other study [27]. This fact becomes relevant because, without coordination, the potential of longitudinally would decrease, compromise comprehensiveness, and the first contact would be essentially administrative. Coordination is defined as a state of harmony resulting from a joint effort. Therefore, it expresses its essence: the availability of information about previous problems and services and its recognition of the service and current needs [38]. Moreover, we did not identify literature that analyses the presence and extent of the PHC set of attributes and the discrepancies between the evaluations of users and professionals through the PCATool in different primary care services. Thus, this study produces relevant and innovative knowledge to support professionals and policymakers in implementing primary health care at the local level by bringing a broader assessment and for the production of knowledge that contributes to the planning of PHC services in the realities of other developing countries.

Comparing our results to the major national epidemiological survey conducted in Brazil (The National Health Survey in 2019) that used the PCAT-Brazil [27], they found an overall score of 5.8[5.6–6.0] for Mato Grosso do Sul State. Our findings have shown an overall score of 5.3 [IC95% 5.2–5.4]. The methodology of the PCAT instrument considers a score ≥ 6.6 as a minimum quality value to assess primary care services from the adult user’s perspective. In both surveys, this general quality value was not reached, confirming that our State is below average in terms of attributes in PHC. Furthermore, it was not seen under professionals’ views that showed values above the minimum of 6.6.

This study has strengths and limitations; the first relates to the instrument (PCAT-Br) that assesses the work process, not precisely the health outcomes in all macroregions. Indeed, through cross-sectional data, we could not infer causality between differences in the work process between all macroregions. Second, as a limitation, the health department’s indication of the PHU and professionals is an important selection bias. However, we agree that the large sample size detects slight mean scores differences and does not influence the results. Third, as we excluded professionals with a temporary contract, it may cause some selection bias in our sample. As potential, we highlight the broad scope of the research, representative of the Mato Grosso do Sul State. To the authors’ knowledge, it is the largest state survey in Mato Grosso do Sul using the full version of the PCAT-Brazil for professionals and users. The instrument used is objective, easy to apply, and presents greater possibilities for comparison since it was evaluated and applied worldwide. In addition, the tool allows the evaluation of the attributes separately, even being related to each other in the health service practice. It is worth emphasising the importance of these attributes positively and concretely. It supports the evaluation and investigation strategies of health systems based on and defined in a service-oriented towards PHC^12^ as the degree of orientation to PHC increased the mental component score of quality of life [38]. Another strength of the study concerns the relationship of inclusion of professionals and users of the same Health Units in the research; most studies developed until then were conducted with professionals or users.

Finally, it is concluded that there is a need to improve the development of PHC in the State, especially the attributes of accessibility and comprehensiveness of the services provided. In addition, it is necessary to strengthen PHC care networks in the State, mainly considering the users’ perspectives.

## Data Availability

The datasets used and/or analysed during the current study are available from the corresponding author on reasonable request.
